# Oral antibiotics and mechanical bowel preparation for colorectal surgery: A prospective observational study of surgical site infection and microbial analysis

**DOI:** 10.1007/s00384-023-04497-4

**Published:** 2023-08-09

**Authors:** Martin Rutegård, Alethea Tang, Darren James Gregoire, Christopher Stewart, Libor Hurt, Susan Chandler, Matthew David Hitchings, Brendan Healy, Dean Harris

**Affiliations:** 1 Department of Colorectal Surgery, Morriston Hospital, Swansea Bay University Health Board, Swansea, UK; 2 Department of Surgical and Perioperative Sciences, Surgery, Umeå University, Umeå, Sweden; 3 Faculty of Medicine, Health and Life Sciences, Swansea University, Swansea, UK; 4 Public Health Wales, Swansea Bay University Health Board, Swansea, UK

**Keywords:** Anastomotic leak, Abscess, Colorectal cancer, Antibiotics, Laxatives, Infection

## Abstract

**Purpose:**

Surgical site infections (SSIs) are common in colorectal surgery. Mechanical bowel preparation (MBP) in conjunction with oral antibiotics (OABs) have been shown to reduce SSI rates. It however is still unclear which OABs to use, and how this can be implemented in practice.

**Methods:**

This is a prospective observational study carried out in Swansea Bay University Health Board during 2019–2021, evaluating the introduction of OABs in a stepwise manner on the incidence of SSI in major colorectal surgery. A control group having MBP only was compared to two OAB groups: one group had MBP plus metronidazole only and the second MBP plus metronidazole and neomycin. A 30-day follow-up after surgery was ascertained via chart review and telephone contact. Logistic regression was performed to estimate the relation between OAB use and SSI, with adjustment for confounding. In a subset of patients, faecal samples were analysed through 16S rRNA amplicon sequencing before and after OAB treatment, depicting the impact of the gut microbiome.

**Results:**

In total 160 patients were analysed: 46 patients had MBP only, whilst 76 patients had MBP plus metronidazole only and 38 patients had MBP with metronidazole/neomycin. The SSI rate in the entire cohort was 33.8%, whilst the adjusted ORs for the single- and dual-OAB groups were 0.76 (95% CI: 0.17–1.81) and 0.50 (95% CI: 0.17–1.52). The microbial analysis demonstrated that the relative abundance for many bacterial genera was changed before and after OAB treatment, but no link with SSI development could be shown.

**Conclusions:**

Introduction of OABs in conjunction with MBP in colorectal surgery is feasible, and may potentially lead to lower rates of SSI, as well as altering the community structure of the faecal microbiome. More research is needed, especially considering different OABs and mechanistic studies of the gut microbiome in the context of colorectal surgery.

## Introduction

Post-operative complications are common after colorectal cancer surgery, of which surgical site infections (SSI) amount to 20–40% [1]. SSIs are a significant cause of morbidity and mortality, especially when due to anastomotic leakage. Anastomotic leaks affect about 10–20% of rectal cancer patients [2, 3] and leaks are associated with cancer recurrence [4].

Current meta-analysis results indicate that a strategy of a short perioperative specific oral antibiotic (OAB) course, so-called selective decontamination, reduces the incidence of SSI and anastomotic leakage [5]. However, broad-spectrum antibiotics perform worse than selective oral antibiotics, suggesting that there is a distinct niche of pathologic bacteria involved in SSI events [6]. Whilst some trials demonstrate that OABs and mechanical bowel preparation (MBP) are effective measures and are included in the World Health Organisation’s SSI guidelines [6, 7], recent randomised clinical trial data examining no MBP at all partly challenge this position [8]. Moreover, whilst some of the recent selective decontamination trials have microbiome data, there have been few attempts at characterizing the pre-operative microbiome—such an approach would potentially offer individualised decontamination measures, such as tailored OABs to a specific microbiome on an individual level. This is likely an important factor, as an emerging body of experimental literature suggests that only particular bacterial strains, e.g. *Enterococcus faecalis* and *Pseudomonas aeruginosa*, play a causative role in the development of anastomotic leakage [9, 10].

With the aim to evaluate the introduction of oral antibiotics and mechanical bowel preparation, a prospective study was initiated to assess the impact of different strategies on SSI rates. In addition, faecal sampling was performed to evaluate the effect on the microbiome of surgery, MBP, and OABs.

## Method

### Checklist for the reporting of observational studies

This article was written in accordance with the Strengthening The Reporting of Observational Studies in Epidemiology (STROBE) checklist for the reporting of observational studies [11].

### Study design

This is a prospective observational study of patients operated in Swansea Bay University Health Board, encompassing two sites. The same team of colorectal consultants were active during the study period. Operations consisted of elective major colorectal surgery, ranging from segmental colon resections to total pelvic exenteration.

The project was reviewed by the Joint Scientific Review Committee for Swansea Bay University Health Board who deemed the project to be service evaluation and non-research based on the use of the 16S sequencing to confirm the effect of the oral antibiotic system change. In agreement with Public Health Wales and Medicines Management Group, single- and dual-antibiotic regimens were formulated and used in a stepwise-addition manner. The choice of OAB was agreed with the microbiology department, and it was agreed that this antibiotic choice should be subject to evaluation and future audit cycles. As anaerobes constitute a significant part of the intestinal microflora, control of anaerobes with an effective antimicrobial was deemed imperative in preventing SSI. Metronidazole was chosen for its effectiveness in anaerobic organisms and was used as monotherapy to counter the risk of induction of *Clostridioides difficile* with dual therapy; moreover, the use of oral metronidazole alone in SSI reduction is sparsely studied. Neomycin and metronidazole were chosen as it is a common combination used in clinical practice for the prevention of SSI in elective colorectal surgery. Secondly, this regimen was chosen so that SSI rates can be compared to other studies and not be confounded by heterogeneity due to antimicrobial choice.

#### Standards and control group

Site-specific baseline figures (using bowel preparation alone) were taken from an all-Wales audit conducted between March 1 and 21, 2019 [12]. Elective colorectal operation data for Swansea Bay University Health Board were extracted and used as a comparative control group. The control group did not receive any OAB. MBP was administered to all colorectal resections based on locally agreed protocols. This comprised two sachets of sodium picosulfate (Picolax®, Ferring Pharmaceuticals Ltd.): one sachet to be taken 2 days prior to surgery and one sachet taken the day before surgery. Each sachet contains 10 mg of the active ingredient sodium picosulfate.

#### Intervention groups and subsequent audit cycles

This study was carried out from July 2019 to March 2021. Patients were divided into two interventional groups and outpatient prescriptions provided for MBP and OAB. MBP comprised two sachets of sodium picosulfate and administered as described above. The first group underwent surgery between July and September 2019 and received MBP and OAB consisting of three doses of metronidazole 400 mg (Crescent Pharma Ltd.) taken at 8 hourly intervals the day before surgery. The second interventional group underwent surgery from January to March 2021 and received MBP and dual OAB, comprising three doses of metronidazole 400 mg (Crescent Pharma Ltd.) and neomycin sulfate 1 g (imported from Teva Pharmaceuticals, Parsippany, NJ, 07054, USA); OAB was administered at 8 hourly intervals the day before surgery. The dosing schedule recommended commences at 10 am, with the two subsequent doses taken at 2 pm and 10 pm. Patients failing to adhere to the full oral pre-operative regimen were excluded. Intravenous prophylactic antibiotics were used for all groups and were standardised based on local antimicrobial guidelines. In general, 1.5 g cefuroxime and 500 mg metronidazole were given on induction, whereas 400 mg of ciprofloxacin was given as an alternative in the presence of penicillin allergy.

To study the effects of OAB on the gut microbiome, faecal samples were collected and preserved using the OMNIgene.GUT OM-200 (DNAgenotek) collection kits prior to starting MBP and OAB and subsequently intra-operatively — from resected colon (if an anastomosis was formed) or from the lumen of the colon during stoma formation. Instructions were given to participants on the use of the collection kits prior to being provided with them for the pre-operative sample. Intra-operative samples were collected by the research team at the time of surgery. The OMNIgene.GUT OM-200 samples were collected, stored, and transported at ambient temperature prior to storage at −80 °C for longer term storage in accordance with the manufacturers’ recommended guidelines and instructions.

All faecal samples were analysed using 16s rRNA amplicon sequencing. For both intervention groups any patients diagnosed with an SSI had microbiological cultures grown from wound and fluid output from intra-abdominal drains.

### Outcome

The primary outcome was any SSI within 30 days of surgery, according to the definition used by United States Centre for Disease Control (CDC) [13]. SSI data were collected according to CDC guidelines [14] from the date of surgery up to 30 days post-operatively via telephone follow-up after discharge from hospital. Active inpatient monitoring of drug charts and recording of additional antibiotics were recorded. Daily clinical reviews, radiology investigations, and microbiology reports were recorded. These were monitored through clinical note review for wound/deep infection. Data capture also include prescription of additional antibiotics for presumed infections, wound swab results, and visits to the general practitioner after hospital discharge. Secondary outcomes included organ/space SSI; anastomotic leak, as defined by an international consensus group [15]; readmission, length of stay (for index admission), and mortality as well as wound cultures and genome sequencing of cultured organisms. All outcomes were measured within 30 days of surgery.

### Statistical analysis of clinical data

Frequency tables concerning patient characteristics, operative details, and intravenous antibiotic use were constructed. Continuous variables were described using the median along with the interquartile range (IQR). Fisher’s exact test was used to test for associations in selected categorical comparisons. Univariable and multivariable binary logistic regression was performed to estimate the association of received oral antibiotics (none, metronidazole only, or metronidazole and neomycin) with any SSI event. To adjust for potential confounding, the following covariates were considered based on the literature on colorectal SSI events: age (years; continuous), sex (male, or female), laparoscopy (no/converted, or yes), use of induction intra-operative antibiotics (no, or yes), and type of operation (right hemicolectomy, left hemicolectomy/sigmoid resection, anterior resection, abdominoperineal excision/Hartmann’s procedure, total pelvic exenteration, or other). Results are reported as odds ratios (OR) with corresponding 95% confidence intervals (CIs). A complete cases analysis was used throughout, as missing data were rare. The analysis was performed in Stata version 16.1 (StataCorp, College Station, TX: StataCorp LLC).

### Microbiome analysis

#### DNA extraction, library preparation, and sequencing

Total DNA was extracted from faecal samples (preserved in the OMNIgene.GUT OM-200 (DNAgenotek) collection kits), using the Qiagen QIAamp PowerFecal Pro DNA Kit. Primers targeting the 16S V4 hypervariable were used to amplify this V4 region as described previously [16]. Library QC and normalisation were carried out using a Qubit HS DNA quantification kit prior to sequencing on an Illumina MiSeq platform at the Swansea University Medical School Sequencing Facility.

#### 16s rRNA amplicon curation and statistical analysis

Sequences were processed using Mothur (version 1.44.1) and Mothur’s SOP guidelines. Sequences were clustered to form operational taxonomic unit (OTUs) using a dissimilarity cut-off of 3%. Classification was carried out using the SILVA release 132 reference with the removal of chloroplast, eukaryote, mitochondrial, or archaeal identified sequences. The **α**-diversity metrics were calculated using Mothur (number of OTUs (*S*_obs_), Shannon evenness, and the inverse Simpson diversity index). Further statistical analysis and graphical representations were completed using RStudio using the tidyverse package (version 1.3.2), phyloseq (version 1.40.0), and vegan (version 2.6.2). Comparisons of **α**-diversity metrics were conducted using a pairwise Wilcoxon rank sum test. Beta-diversity comparisons were made by calculating the Bray-Curtis distance between samples at OTU level together with non-metric multidimensional scaling (NMDS) calculation for visualisation purposes. Analysis of variance between pre- and post-antibiotic treatment communities and between SSI and non-SSI communities was explored using the adonis2 (PERMANOVA) function in vegan (version 2.6.2) using the Bray-Curtis distance matrix and 10,000 permutations. Differences in abundance of taxa between pre-treatment samples and neomycin- and metronidazole-treated samples were identified at genus level, using a pairwise Wilcoxon rank sum test approach. Multiple comparisons were corrected for by applying the Benjamini-Hochberg false discovery rate controlling procedure.

## Results

### Patients

**Fig. 1:**
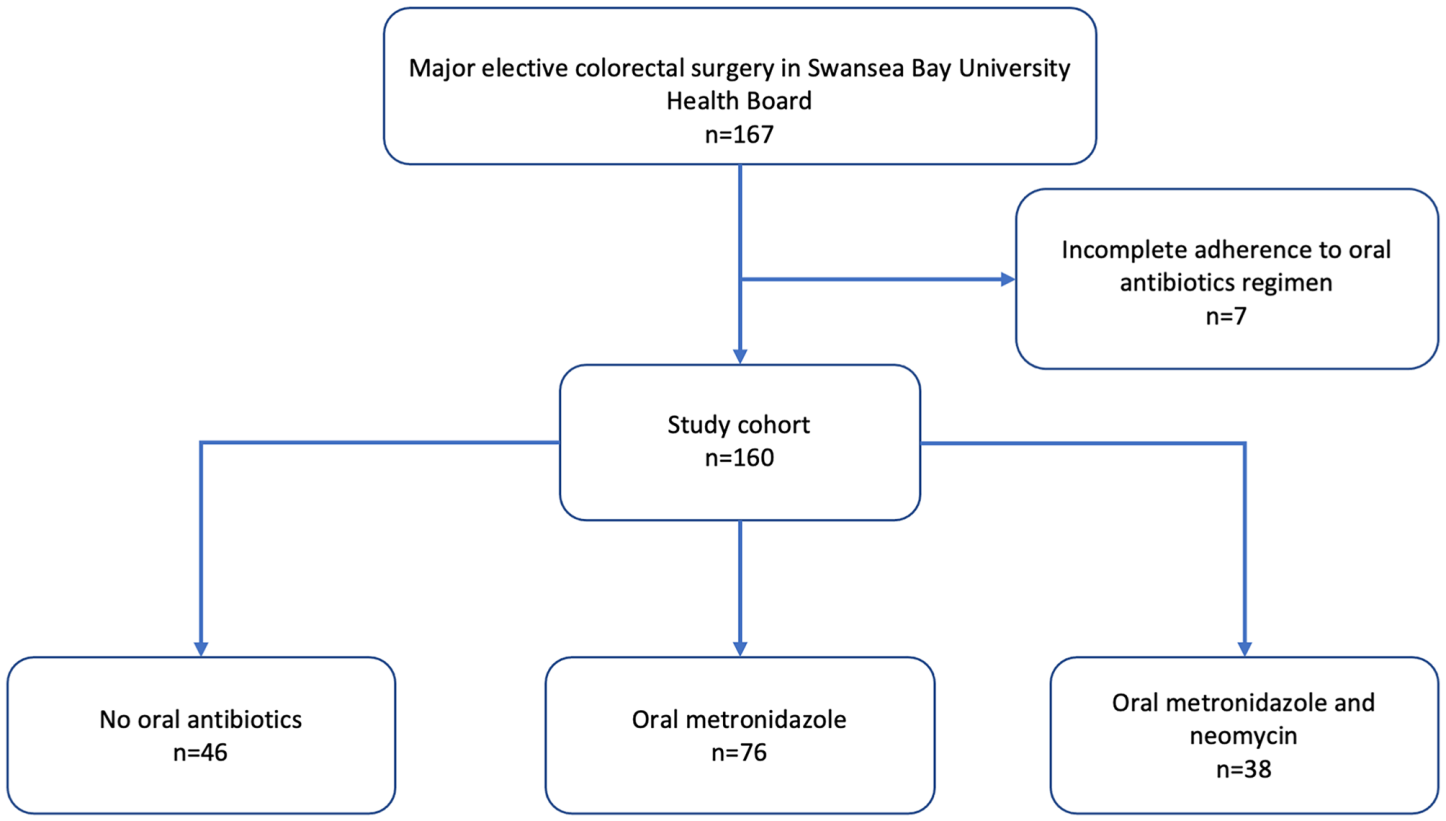
Study flowchart

During the study period, 167 patients were included, whereas seven patients were excluded due to incomplete adherence to the prescribed OAB regimens. All patients complied to MBP instructions. Of the 160 analysed patients, 46 patients had MBP only, 76 patients had metronidazole, and 38 patients had metronidazole and neomycin (Fig. 1). The demographic and clinical variables are presented in Table 1, stratified by received OAB. Patients in each group were similarly distributed in respect to sex and operation type, whilst patients in the metronidazole/neomycin were younger and had more laparoscopic surgery. The use of induction intra-operative antibiotics was slightly less common in the OAB groups. All participants were successfully followed up to 30 days as intended, through a combination of telephone follow-up and medical notes (including recent attendances and prescriptions in primary care, if any).

**Table 1:** Baseline characteristics for 160 patients operated in Swansea Bay University Health Board, stratified by received oral antibiotics

	**None** (*n* = 46)	**Metronidazole** (*n* = 76)	**Metronidazole/neomycin (***n* = 38)
**Age (years)**	68.0 (53.0–75.0)	70.0 (56.5–76.5)	64.5 (53.0–77.0)
**Sex**			
Male	24 (52.2%)	41 (53.9%)	21 (55.3%)
Female	22 (47.8%)	35 (46.1%)	17 (44.7%)
**Operation type**			
Right hemicolectomy	16 (34.8%)	17 (22.4%)	12 (31.6%)
Left/sigmoid hemicolectomy	5 (10.9%)	9 (11.8%)	5 (13.2%)
Anterior resection	5 (10.9%)	24 (31.6%)	12 (31.6%)
APER/Hartmann/proctectomy	12 (26.1%)	21 (27.6%)	8 (21.1%)
Total pelvic exenteration	4 (8.7%)	3 (3.9%)	1 (2.6%)
Other	4 (8.7%)	2 (2.6%)	0 (0.0%)
**Laparoscopy**			
Open/converted	37 (80.4%)	58 (76.3%)	17 (44.7%)
Laparoscopy	9 (19.6%)	17 (22.4%)	21 (55.3%)
Missing	0 (0.0%)	1 (1.3%)	0 (0.0%)
**Intra-operative antibiotics**			
No	1 (2.2%)	14 (18.4%)	10 (26.3%)
Yes	45 (97.8%)	62 (81.6%)	28 (73.7%)
**Surgical site infection**			
None	27 (58.7%)	50 (65.8%)	29 (76.3%)
Superficial	12 (26.1%)	16 (21.1%)	6 (15.8%)
Deep	1 (2.2%)	1 (1.3%)	1 (2.6%)
Organ/space	6 (13.0%)	9 (11.8%)	2 (5.3%)
**Length of stay (days)**	8.0 (6.0–12.0)	8.0 (5.5–12.5)	6.0 (4.0–8.0)
**Readmission**			
No	39 (84.8%)	66 (86.8%)	36 (94.7%)
Yes	7 (15.2%)	10 (13.2%)	2 (5.3%)
**Post-operative mortality**			
No	43 (93.5%)	76 (100.0%)	38 (100.0%)
Yes	3 (6.5%)	0 (0.0%)	0 (0.0%)

Data are presented as median (IQR) for continuous measures and *n* (%) for categorical measures

*APER* abdominoperineal excision of the rectum

### Surgical site infection

The SSI rate in the entire cohort was 54/160 (33.8%). The SSI events were mostly superficial (63.0%) in nature, organ/space SSI was the next most common (31.5%), whilst deep SSI was rarer (5.6%). The SSI rates were highest in the no antibiotic group at 19/46 (41.3%), the metronidazole group at 26/76 (34.2%), and metronidazole/neomycin group at 9/38 (23.7%). The dual-OAB group had the lowest rate of SSI, although not statistically significantly so (Fisher’s exact *p* = 0.231). Using no antibiotics as reference, the adjusted ORs of metronidazole and metronidazole/neomycin were 0.76 (95% CI: 0.17–1.81) and 0.50 (95% CI: 0.17–1.52). The sequential reduction in SSI risk with OAB is suggestive of a treatment effect but statistical significance was not reached (Table 2).

**Table 2:** The risk of developing a surgical site infection (SSI) within 30 days of major colorectal resection in Swansea Bay University Health Board, as a function of received oral antibiotics 24 h before surgery

**Received per oral antibiotics**	**Any SSI (%)**	**Unadjusted OR (95% CI) for SSI**	**Adjusted**^**a**^ **OR (95% CI) for SSI**
**None**	19/46 (41.3)	Reference	Reference
**Metronidazole**	26/76 (34.2)	0.74 (0.35–1.57)	0.76 (0.32–1.81)
**Metronidazole/neomycin**	9/38 (23.7)	0.44 (0.17–1.14)	0.50 (0.17–1.52)

Odds ratios (ORs) and 95% confidence intervals (CIs) are estimated with logistic regression, without and with adjustment for confounding

^a^With adjustment for age, sex, laparoscopy, induction antibiotic use, and type of operation

### Secondary outcomes

In regard to the secondary outcomes, organ/space SSI was least common in the metronidazole/neomycin group at 5.3%, but further analysis was precluded by the small sample size. Similar trends were noted with a readmission rate in the latter group at 5.3%, whereas length of stay was also shortest with 6 days. Anastomotic leakage was detected in seven patients; all underwent an anterior resection, with a resulting rate of 17.1%. No leaks were noted in the no antibiotic group; no statistical differences were noted (Fisher’s exact *p* = 0.844). Conversely, post-operative mortality at 6.5% was only noted in the no antibiotic group.

### Microbial analysis

**Fig. 2:**
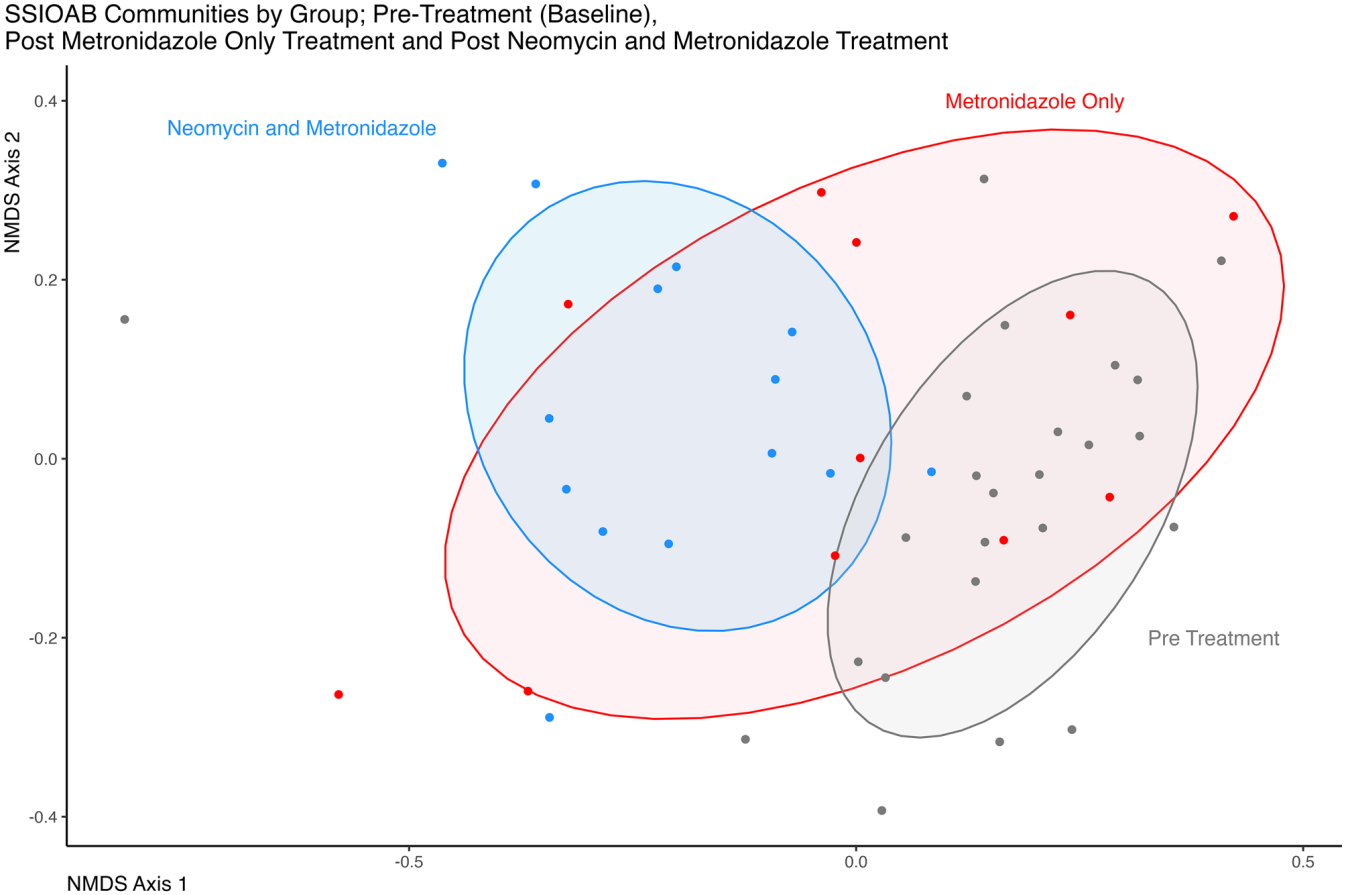
Bray–Curtis distances based non-metric multidimensional scaling (NMDS) (stress 0.18) of samples belonging to the pre- and post-treatment groups. Permutational multivariate analysis of variance (PERMANOVA) highlights that the microbial communities differ significantly between the pre- and intra-operative samples when neomycin and metronidazole are used (corrected *p* = 0.003) but not when metronidazole only is used (corrected *p* = 0.809). Significant differences in microbial communities were also observed between intra-operative sample groups metronidazole only and neomycin and metronidazole (corrected *p*-value = 0.003)

**Fig. 3:**
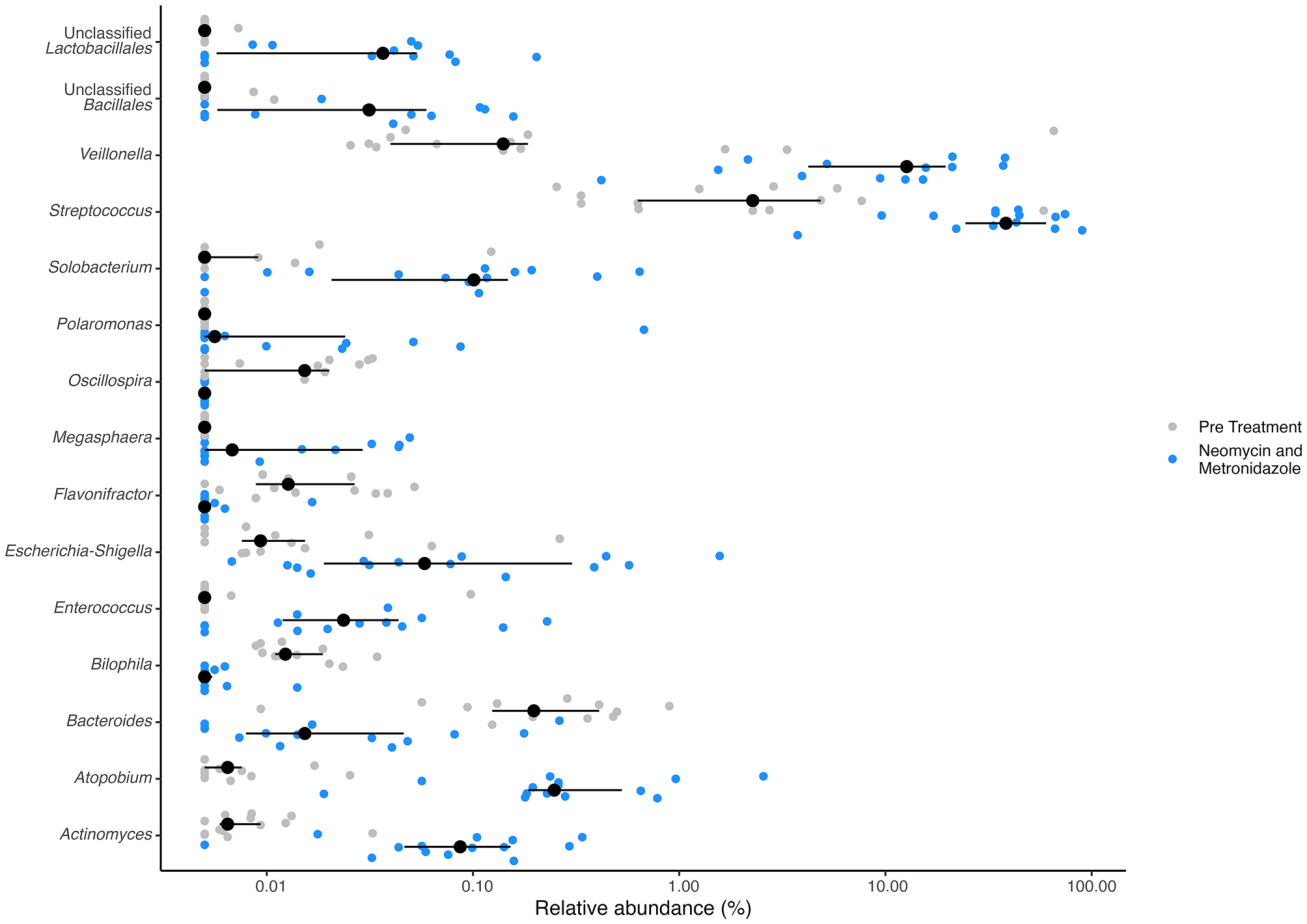
Differentially abundant taxa detected between pre-treatment and treatment with neomycin and metronidazole, where both groups have been subjected to mechanical bowel preparation. Blue and grey dots represent the relative abundance of the noted taxa at genus level on a per sample basis. Black dots and lines represent median, lower, and upper quartiles

To elucidate if gut microbiota is related to SSI rates, we sequenced faecal samples using the V4 region of the 16 S rRNA gene. A total of 13 patients provided faecal samples in the neomycin and metronidazole group and 11 patients in the metronidazole-only group, with faecal samples both pre-antibiotic administration and intra-operatively. Receipt of antibiotics did not significantly alter the richness, evenness, or diversity of gut bacterial communities (corrected *p* > 0.05 for all pairwise comparisons). Community structure was assessed by exploring the Bray-Curtis metric at OTU level (Fig. 2). Intra-operative faecal samples displayed a significant difference in the bacterial community structure compared to pre-operative samples when neomycin and metronidazole were used (corrected *p* = 0.003). There was no significant difference in bacterial community structure between pre- and intra-operative samples in the metronidazole-only group (corrected *p* = 0.809). However, when comparing intra-operative sampled communities between the two antibiotic groups (metronidazole only vs neomycin and metronidazole), a significant difference in community structure was observed (corrected *p* = 0.003). To explore the taxa responsible for the observed difference between the pre- and post-operative samples from the neomycin and metronidazole group, a pairwise Wilcoxon rank sum test was conducted. A total of 15 taxa were identified as having differential abundance between the communities of the two sampling points within the neomycin and metronidazole group (corrected *p* < 0.05) (Fig. 3) The corresponding mean relative abundance values and *p* values are presented in Table 3. Despite the observed compositional differences between the antibiotic-treated samples, there were no significant differences in the bacterial communities sampled pre- or intra-operatively in those who developed an SSI (*n* = 5) compared to those who did not develop an SSI (*n* = 19) (corrected *p* = 0.9).

**Table 3:** Differentially abundant taxa detected between pre-treatment and treatment with neomycin (N) and metronidazole (M), where both groups have been subjected to mechanical bowel preparation

**Taxonomy**	**Corrected** ***p*** **value**	**Mean relative abundance pre-treatment**	**Mean relative abundance after N&M**
***Actinomyces***	0.00599	0.00954	0.11294
***Atopobium***	0.00197	0.00838	0.48951
***Bacteroides***	0.01377	0.28737	0.05117
***Bilophila***	0.00245	0.01518	0.00588
***Enterococcus***	0.02525	0.01226	0.04642
***Escherichia-Shigella***	0.04577	0.03424	0.24547
***Flavonifractor***	0.01377	0.01912	0.00596
***Megasphaera***	0.04577	0.00500	0.01785
***Oscillospira***	0.01410	0.01511	0.00500
***Polaromonas***	0.04577	0.00500	0.06511
***Solobacterium***	0.02287	0.01603	0.14162
***Streptococcus***	0.00245	6.78528	41.6682
***Veillonella***	0.00599	5.49284	14.0288
***Bacillales unclassified***	0.02263	0.00573	0.04598
***Lactobacillales unclassified***	0.01377	0.00518	0.04509

## Discussion

In this prospective observational study of sequential introduction of OAB for major colorectal surgery, SSI reductions were suggested with the antibiotic combination of metronidazole and neomycin. Statistical significance was not attained, possibly due to the limited sample size. The faecal microbiome analysis of the bacterial community revealed that the combination of metronidazole and neomycin affected the community structure when comparing faecal samples before surgery from intra-operative samples, whilst administration of metronidazole alone did not lead to such changes. Despite there being no significant difference between communities of those who developed an SSI compared to those who did not, identification of taxa that differentiate communities after metronidazole and neomycin introduction may provide a biological mechanism for a potential SSI reduction. When considering the contribution to disease and overall gut health, a change in the 15 taxa that displayed differential abundance in the metronidazole and neomycin group could be considered to have a net beneficial effect. *Bacteroides* spp. and *Flavonifractor* have been reported to be positively associated with tumorigenesis and colorectal cancer [17, 18], and the mean relative abundance of both were decreased within the metronidazole and neomycin group. Similarly, abundance changes of *Bilophila* have been associated with inflammatory responses in both mice and humans [19]. The increased abundance of the taxa *Megasphaera, Polaromonas, Streptococcus, Veillonella*, and *Lactobacillales* presented within have also all been reported to positively correlate with good gut health. The short-chain fatty acids producing *Megasphaera* have demonstrated a protective role against diarrheal cryptosporidiosis [20] whilst good responders following bariatric surgery were characterised as having increased abundances of *Polaromonas* [21]. Lactic acid bacteria of *Lactobacillales* are reported as being important in maintenance of the intestinal barrier [22], making their putative increase in relative abundance subsequent to metronidazole and neomycin treatment worthy of further exploration. Despite there being demonstrable positive changes in taxa associated with disease and protection thereof, *Actinomyces* and *Escherichia-Shigella* were found to have increased proportions. Both taxa have been associated with disease development and progression, as *Actinomyces* positively correlated with pro-tumour microbial taxa in young-onset colorectal cancer [23] and *Escherichia-Shigella* was related to nephropathy [24]. As such, the full extent to which the antibiotic treatment permits improvement in gut health as measured by decrease in SSI rates requires further exploration; limitations within this study, particularly with regards to cohort size, prevent robust analysis of their short- and long-term effects.

The small sample size allowed confounder adjustment only to a minor degree. Furthermore, residual confounding is almost certain in this study, due to lack of information on comorbidity and the non-randomised allocation of the exposure OAB. Nevertheless, the OAB was introduced in a stepwise manner for all patients in each study period, without the involvement of the surgeons performing the operations, and one could argue that confounding might therefore be limited. The only minor changes of the point estimates after adjustment for covariates in the main analyses may be a testament to this. Another weakness includes the telephone follow-up to ascertain SSI diagnosis, as this is not a validated tool. There was also no check on diagnostic accuracy of community SSIs, and this relied upon general practitioner or patient interpretation. However, many studies do not count SSI outside of hospital, in contrast to the 30-day follow-up in both primary and secondary care of the present study. Yet another difficulty when interpreting the study results is the potential impact of the coronavirus pandemic, as all the patients in the dual-OAB group were admitted through a green pathway during this period, with an increased emphasis on hygiene and using protective equipment; there is data showing that such pathways decrease pulmonary complications and associated mortality, whilst the impact on surgical SSIs is less clear [25].

There is a strong trend in contemporary colorectal surgery to employ the tenets of enhanced recovery of surgery (ERAS). In 2018, the ERAS society issued guidelines concerning OAB and MBP based on the literature, recommending against MBP alone as this imparts fluid losses and electrolyte disturbances without any benefit; however, OAB and MBP in combination could be considered to reduce SSIs, albeit with a weak evidence base [26]. The current study arm with MBP only demonstrated the highest SSI incidence, in line with these previous findings. Since these guidelines were published, major achievements have been made with several randomised clinical trials. A Spanish trial compared ciprofloxacin and metronidazole with placebo the day before surgery in colon surgery, where a difference of 11 vs 5% was noted in favour of the OAB arm [27]. This study was conducted without MBP and, in comparison to the present study, with less complex operations, but it bolsters the concept of OAB to reduce SSIs. Interestingly, in a Finnish trial on colon resections comparing MBP in combination with a single-dose metronidazole and neomycin the day before surgery with no pre-operative treatment, SSI rates were not significantly different at 7 versus 11% [8]. Whilst this study might be underpowered, differences with the current study cohort could also be ascribed to a control arm without MBP and the single-dose regimen used, perhaps not sufficient to impact the gut microbiome. A French trial comprising colorectal resections compared ornidazole only versus placebo and found an SSI difference of 22 vs 13%, whilst the subset of patients with MBP had an even greater benefit from OAB [28]. Whilst strengthening the hypothesis that OAB and MBP in combination are beneficial, in the current study clear SSI improvements were suggested only in the dual-OAB group, which might be explained by the admittedly weaker study design. To conclude, a contemporary meta-analysis including most but not all of the above studies as well as others suggest that OAB and MBP in combination confer substantial reductions in SSI rates [6]. In an interesting subanalysis, it was reported that broad-spectrum OAB in contrast to more selective OAB did not seem to impart such SSI reductions. Concerning the present study, this could explain the apparent gradient from higher to lower SSI rates in the groups without OAB, to a broad-spectrum OAB such as metronidazole, to a combination of a broad-spectrum and selective OAB, i.e. neomycin.

The biological underpinnings of an effect of OAB, especially of the selective variety (e.g. aminoglycosides), can be derived from experimental data suggesting that only some bacterial strains, e.g. *Enterococcus faecalis* and *Pseudomonas aeruginosa*, play a causative role in the development of anastomotic leakage [9, 10], which is a major cause of SSI in colorectal surgery. The dual OABs in this study seemed to reduce SSIs as well as to impart differences in the gut microbiome, including increasing the relative abundance of *Enterococcus*. However, the exact serotype is not known and at this stage no conclusions can be drawn regarding any mechanistic links from the current study, not the least since no differences concerning the microbiome could be detected when comparing patients developing SSI with those who did not. Nevertheless, future research could, based on the previous evidence base on selective OABs and potential causative organisms, include providing neomycin or vancomycin only or both in combination. A stepwise approach as implemented in the present study is a practical design reflecting real-world implementation and practice but might be prone to unforeseen confounding and ideally such research should be performed using randomised designs.

In conclusion, the combination OAB metronidazole and neomycin with MBP before major colorectal surgery seems to be related to a decrease in SSI within the first 30 post-operative days, though statistical significance was not attained, perhaps due to limited sample size. The impact of the OAB regimen could be discerned on the impact of the community structure of the gut microbiome, whilst a clear link to SSI development could not be demonstrated. These findings suggest a potential role for OABs in reduction of SSI, though more research with larger cohorts and trials is needed to determine the effects of particular OABs, individually and in combinations.

## Data Availability

No material from other sources was included in the article.

## Data availability

Upon reasonable request, data can be shared, subject to approval from Swansea Bay University Health Board. Raw sequence reads have been deposited in the NCBI SRA database and can be accessed through the NCBI BioProject accession number PRJNA991438.
